# Hospital Accounts and Finance

**Published:** 1923-08

**Authors:** Joseph E. Stone

**Affiliations:** (Accountant, St. Thomas's Hospital)


### HOSPITAL ACCOUNTS AND FINANCE,

### A NEW SYSTEM OF ACCOUNTING CONTROL.

By JOSEPH E. STONE, F.S.A.A., F.S.S., F.R.Econ.S. (Accountant, St. Thomas's Hospital).

UNDOUBTEDLY the most significant test of the efficiency of hospital activities is the value of the services rendered in terms of pounds spent—that is to say, we must gain the proper relation between expenses and the services obtained by such expenses. This is the basis of all economical spending. An increase in expenditure is not itself an indication of lack of efficiency or lack of economy. Every new function assumed by a hospital carries with it an increase in expenditure covering the cost of the new function. An increase in the activities of a special department may lead to a growth in the expenditure, but the service resulting therefrom through the decrease in ill-health and perhaps death, may more than compensate for the extra cost. The creation of a department to assist the management in controlling the finances would increase the expenditure under certain headings, but it would, if energetically supported, lead to real economy and more efficient administration. An increase in expenditure comparatively may indicate that there has been an increase in the effectiveness of the discharge of the hospital's activities. On the other hand, a decrease in expenditure may not represent efficiency or economy in any sense. Expenditure may be decreased to the positive detriment of the services rendered. It is thus obvious that the services resulting from hospital activities cannot be reflected in the accounts as now presented, and supplementary data must be obtained if use is to be made of the accounts as a check on the administration. This supplementary data may take various forms; at present we have the average cost per occupied bed, the cost per out-patient, and the cost per out-patient attendance. But it is now generally agreed that these statistics do not give sufficient information upon which to judge efficiency or economy.

### Accounting by Services.

The remedy lies in the adoption of a system of accounts which will show the cost of services. Under the existing system all expenditure is segregated and analysed so as to fall under specified main heads and sub-heads indicating only on what the money has been spent. The income and expenditure accounts show this very clearly, but there the tale of their virtue ends. They do not go very far in the elucidation of the problems of management because they do not show who is responsible for the various items of expenditure, the cost of any of the services rendered by the hospital, or any figures which will act as a guide as to comparative economy in the various wards, &c.; and, lastly, they do not lend themselves to the establishment of certain units of costs in respect of services, so that a more efficient control may be exercised and valuable comparisons made, one service with another and with similar services at other hospitals.

### Classification of Expenditure.

Expenditure can be classified in two ways—according to its nature (as at present) and according to its purpose. The facility with which charges can be easily and accurately classified, if classification is by nature and not by purpose, no doubt accounts for the retention of the present system. The assistant entering the books cannot tell for what services articles will be used, but he can see from the invoices the nature of the purchases, and has no difficulty in classifying them under their appropriate headings and sub-headings. But the accounts show then only the total cost of each kind of thing purchased—dressings, provisions, uniforms, &c. They do not show the cost of the services for the purposes of which these things were bought. The time has undoubtedly arrived when those responsible for the efficient and economical administration of hospitals should consider it more in keeping with modern ideas of control to have expenditure records according to purpose rather than nature, so that the costs of services may be ascertained. The accounts should be viewed from the standpoint of hospital administration rather than from that of hospital bookkeeping. They should not be regarded as an end in themselves, but as an aid for enabling executives to control. This surely is the main purpose of accounts.

### Principles to be Observed.

For this to be done it is essential that the accounts should be reconstructed on the following principles:—1. A separate and comprehensive account should be set up for each main purpose of expenditure, and within such account the detailed expenditure should be grouped according to its nature. 2. Units of cost should be determined by which the actual expenditure on particular purposes might be controlled. 3. Expenditure should be dealt with on a commercial basis (that is, on a basis of "income and expenditure" instead of on the basis of "cash receipts and payments"), representing the true aggregate cost of the services of the year, the increase or decrease in stores held, outstanding assets and liabilities, &c. Each of these principles could well form the subject of a separate article, and it is only intended to touch upon them very briefly here.

### Costs by Services.

No. 1 is of prime importance. With complete and accurate costs of all the various services rendered by a hospital information of the greatest value would be to hand. The accounts would then represent something more than a mere summary of totals, and all artificial attempts to apportion expenditure as between in-patients and out-patients would become unnecessary. With such accounts it should be possible to study not only the all-in cost of treatment, but the cost of each of the separate stages and as they would enable the actual effect of the financial transactions to be readily perceived, they would provide an instrument of control not attainable under the present highly unsatisfactory system. With regard to No. 2, those units for which the costs are figured should be determined solely by the nature of the services rendered. The occupied bed is the unit at present in use for measuring the cost of treatment, and, generally speaking, it is the one used for testing the efficiency and economical administration of a hospital.

### Determination of the Units.

This unit, however, is a most unsatisfactory one; it cannot be relied upon, and as for comparative purposes so many other considerations have to be taken into account, it is practically worthless. Certainly a large number of items of expenditure may be shown for the unit of the occupied bed, but even here distinctions must be made, as, for example, between ordinary ward patients and private room patients. Many items of expenditure, however, are not useful statistically on this unit. Ambulance service, for instance, is the same for a patient brought to the hospital for a two days' stay as for one brought for a two months' stay. Again, not usually are twice as many laboratory tests or examinations, &c., made for a patient woo stays a month as for one who stays only half that time. The units then must be fixed according to the purpose served by the expenditure. The following are suggestive only:—Per occupied bed for wards; per operation for theatres; per examination and per treatment for X-ray department; per mile for ambulance, &c. These averages, which are radically different from each other, are lost to sight in the present cost per bed, which is taken over the whole of the expenditure.

### Importance of Income and Expenditure System.

The third principle involves a fundamental change in the accounting system. The early records of hospitals were designed to give information with reference only to cash receipts and payments. With the increase in knowledge of modern methods of accounting it is unnecessary to explain the inadequacy of such records as a basis of administrative control. It is not intended to depreciate the necessity of a hospital having information as to its cash position, but it is desired to emphasise that to know the cash position only is not sufficient. Knowledge of the true financial position is required, and this can only be obtained from accounts kept on the income and expenditure system. For trustworthy comparisons to be rendered possible all hospital accounts must be kept on this system. The average cost per patient in two hospitals, one working on a cash system and the other on income and expenditure, is not capable of comparison. In the former case it is obviously not the true cost per patient, but is really only the cash paid during the year per patient resident during the year, a very different thing from the cost per patient. To attain the ends here outlined requires a development in organisation and accounting control, and to emphasise this truth is the primary object of this article.

---

### The East Surrey Hospital.

The East Surrey Hospital is the new name for the familiar institution hitherto known as the Reigate and Redhill. Beginning as a cottage hospital, it moved and grew, and the further extension in prospect will give the hospital a size more proportionate to the area that it serves.

